# 4-Chloro-2-[1-(4-ethyl­phen­yl)-4,5-diphenyl-1*H*-imidazol-2-yl]phenol

**DOI:** 10.1107/S2414314619016900

**Published:** 2020-01-03

**Authors:** K. N. Shraddha, S. Devika, Noor Shahina Begum

**Affiliations:** a Department of Studies in Chemistry, Bangalore University, Jnana Bharathi Campus, Bangalore 560 056, Karnataka, India; Purdue University, USA

**Keywords:** crystal structure, imidazole, hydrogen bonding

## Abstract

In the title compound, C_29_H_23_ClN_2_O, the 5-chloro­phenol ring and the imidazole ring are nearly coplanar, with a dihedral angle of 15.76 (9)° between them. The ethyl­phenyl ring and the two phenyl rings subtend angles of 71.09 (7), 43.95 (5) and 36.53 (9)°, respectively, with the imidazole plane. An intra­molecular O—H⋯N hydrogen bond supports the mol­ecular conformation, and an inter­molecular C—H⋯O inter­action, originating from an *ortho*-phenyl H atom, stabilizes the packing arrangement. In addition, a weak C—H⋯π inter­action, also involving an *ortho*-phenyl H atom, is observed.

## Abstract 2

In the title compound, the 5-chloro­phenol ring and the imidazole ring are nearly coplanar, with a dihedral angle of 15.76 (9)°. The ethyl­phenyl ring and the two phenyl rings subtend at angles of 71.09 (7), 43.95 (5) and 36.53 (9)°, respectively, with the imidazole plane.

## Graphical abstract

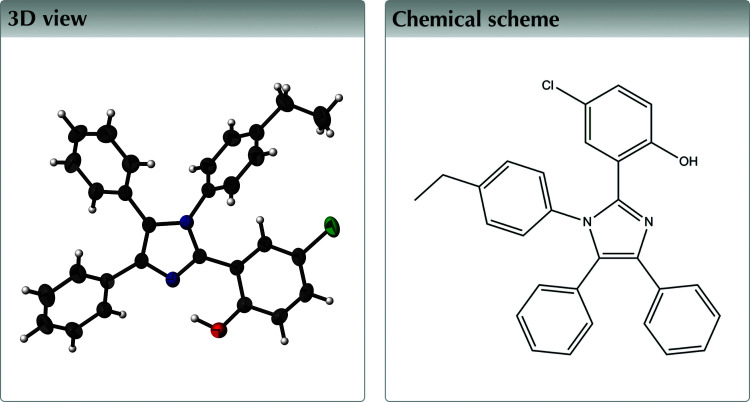

## Structure description

**Figure 1:**
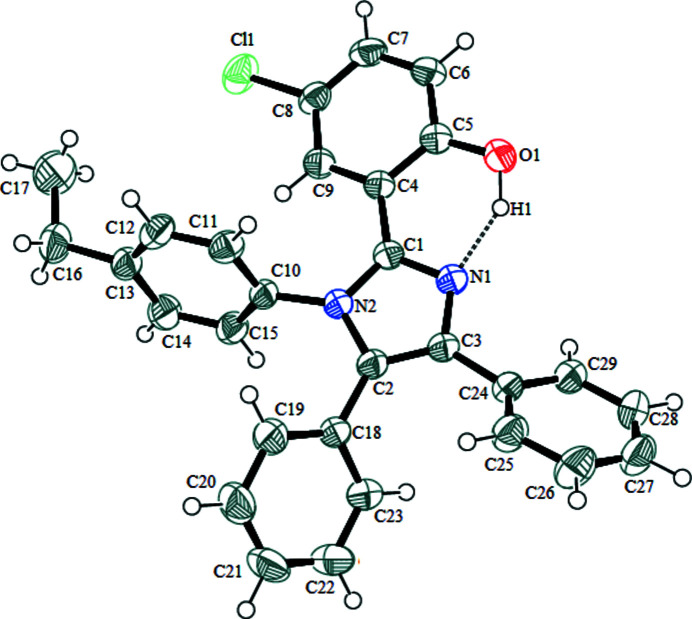
The mol­ecular structure of the title compound with the atom-numbering scheme. Displacement ellipsoids are drawn at the 50% probability level. H atoms are represented as small spheres of arbitrary radius.

**Figure 2:**
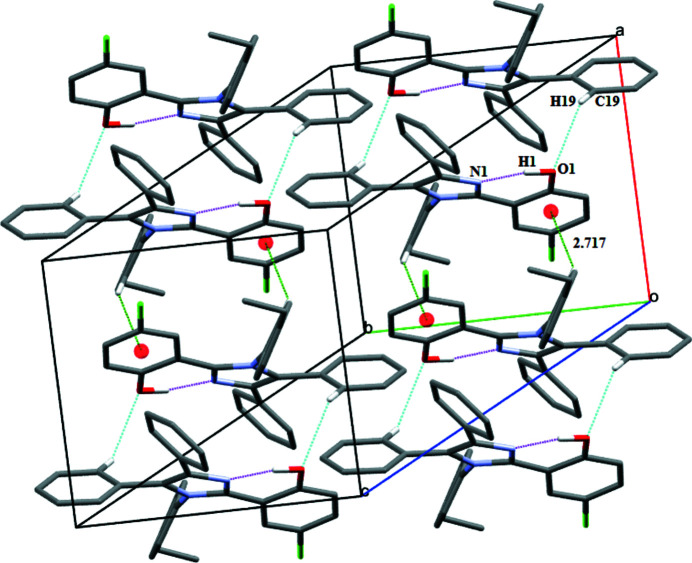
Unit-cell packing of the title compound showing the intra­molecular O—H⋯N inter­actions, inter­molecular C—H⋯O inter­actions and inter­molecular C—H⋯π inter­actions as dotted lines. H atoms not involved in hydrogen bonding have been excluded.

The imidazole moiety is known to play an important role in biological systems being a part of the histidyl residue in peptides and proteins (Sigel *et al.*, 2000 ▸). Multi-substituted imidazoles are an important class of heterocyclic compounds that exhibit diverse biological activities such as anti-inflammatory (Gaonkar *et al.*, 2009 ▸), anti­leishmanial (Bhandari *et al.*, 2010 ▸) and anti­cancer (Ozkay *et al.*, 2010 ▸) activities. As part of our ongoing studies in this area, we herein report the synthesis and crystal structure of the title compound, 4-chloro-2-(1-(4-ethyl­phen­yl)-4,5-diphenyl-1*H*-imidazol-2-yl)phenol (Fig. 1 ▸). The 5-chloro­phenol ring, two phenyl rings and the ethyl­phenyl ring are substituents on the central five-membered imidazole ring (C1/N2/C3/C2/N1). The imidazole and the 5-chloro­phenol rings are close to coplanar with a dihedral angle of 15.76 (9)° between them. The imidazole ring subtends at dihedral angles of 71.09 (7), 43.95 (5) and 36.53 (9)° with the ethyl­phenyl ring and the two phenyl rings (C18–C23 and C24–C29), respectively. A strong intra­molecular O1—H1⋯N1 hydrogen bond is formed between the O1 atom of the 5-chloro­phenol group and atom N1 of the imidazole ring (Fig. 2 ▸), forming an 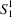 (6) graph-set motif, which stabilizes the close to coplanar arrangement of the imidazole and phenol rings.

In the crystal, atom C19 of the phenyl ring and the hydroxyl O1 atom of the phenol group are involved in a weak C19—H19⋯O1^i^ inter­action that links the mol­ecules along the *a-*axis direction (Fig. 2 ▸). Thus the hydroxyl O atom acts as both a hydrogen-bond donor and an acceptor. The crystal structure is further consolidated by a C15—H15⋯*Cg* ^ii^ inter­action with the aryl ring (Table 1 ▸, Fig. 2 ▸).

## Synthesis and crystallization

The title compound was synthesized by the one-pot reaction of benzil (10 mmol), 4-ethyl­aniline (10 mmol) and 5-chloro-2-hy­droxy­benzaldehyde (10 mmol) with ammonium acetate (10 mmol) in a glacial acetic acid (20 ml) medium. The mixture was refluxed for 5 h at 343 K, the progress of the reaction being monitored by TLC. After completion of the reaction, the mixture was cooled to room temperature and poured into 100 ml of ice-cold water. The resulting precipitate was filtered, dried and further purified by column chromatography (7:3 petroleum ether:ethyl acetate) and isolated in good yield (85%). The product was recrystallized from ethanol solution. IR (KBr) (cm^−1^): 3448.63 (OH), 1947.51 (C=C), 1601.84 (C=N). ^1^H NMR (CDCl_3_): δ 1.242 (*t*, *J* = 7.2 Hz, 2H), 2.64–2.68 (*q*, *J* = 7.2 Hz, 3H), 7.09–7.26 (*m*, 13H), 7.50–7.52 (*m*, 4H), 6.34–6.35 (*s*, 1H). GC–MS (EI, 70 eV): *m*/*z*: 450.95.

## Refinement

Crystal data, data collection and structure refinement details are summarized in Table 2 ▸.

## Supplementary Material

Crystal structure: contains datablock(s) global, I. DOI: 10.1107/S2414314619016900/zl4037sup1.cif

Structure factors: contains datablock(s) I. DOI: 10.1107/S2414314619016900/zl4037Isup2.hkl

Supporting information file. DOI: 10.1107/S2414314619016900/zl4037sup3.tif

Supporting information file. DOI: 10.1107/S2414314619016900/zl4037sup4.pdf

Proton-NMR. DOI: 10.1107/S2414314619016900/zl4037sup5.pdf

Supporting information file. DOI: 10.1107/S2414314619016900/zl4037Isup6.cml

CCDC reference: 1950453

Additional supporting information:  crystallographic information; 3D view; checkCIF report

## Figures and Tables

**Table 1:** Hydrogen-bond geometry (Å, °) *Cg* is the centroid of the C4–C9 aryl ring.

*D*—H⋯*A*	*D*—H	H⋯*A*	*D*⋯*A*	*D*—H⋯*A*
O1—H1⋯N1	1.00	1.66	2.549 (1)	145
C19—H19⋯O1^i^	0.93	2.57	3.242 (3)	129
C15—H15⋯*Cg* ^ii^	0.93	2.72	3.527 (2)	146

Symmetry codes: (i) 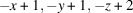 ; (ii) 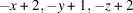 .

**Table 2:** Experimental details

Crystal data	
Chemical formula	C_29_H_23_ClN_2_O
*M*_r_	450.94
Crystal system, space group	Monoclinic, *P*2_1_/*n*
Temperature (K)	297
*a*, *b*, *c* (Å)	9.0627 (6), 10.7595 (8), 24.4636 (19)
β (°)	100.599 (3)
*V* (Å^3^)	2344.7 (3)
*Z*	4
Radiation type	Mo *K*α
μ (mm^−1^)	0.19
Crystal size (mm)	0.45 × 0.38 × 0.35

Data collection	
Diffractometer	Bruker SMART APEX CCD
Absorption correction	Multi-scan (*SADABS*; Bruker, 1998 ▸)
*T*_min_, *T*_max_	0.821, 0.928
No. of measured, independent and observed [*I* > 2σ(*I*)] reflections	34596, 4807, 3593
*R*_int_	0.044
(sin θ/λ)_max_ (Å^−1^)	0.627

Refinement	
*R*[*F* ^2^ > 2σ(*F* ^2^)], *wR*(*F* ^2^), *S*	0.047, 0.129, 1.03
No. of reflections	4807
No. of parameters	301
H-atom treatment	H atoms treated by a mixture of independent and constrained refinement
Δρ_max_, Δρ_min_ (e Å^−3^)	0.26, −0.29

Computer programs: *SMART*and *SAINT-Plus* (Bruker, 1998 ▸), *SHELXS97* (Sheldrick, 2008 ▸), *SHELXL2018* (Sheldrick, 2015 ▸) and *ORTEP-3 for Windows* (Farrugia, 2012 ▸).

## full crystallographic data

### Crystal data

**Table:**

C_29_H_23_ClN_2_O	*F*(000) = 944
*M**_r_* = 450.94	*D*_x_ = 1.277 Mg m^−^^3^
Monoclinic, *P*2_1_/*n*	Mo *K*α radiation, λ = 0.71073 Å
*a* = 9.0627 (6) Å	Cell parameters from 4807 reflections
*b* = 10.7595 (8) Å	θ = 2.6–26.5°
*c* = 24.4636 (19) Å	µ = 0.19 mm^−^^1^
β = 100.599 (3)°	*T* = 297 K
*V* = 2344.7 (3) Å^3^	Block, colorless
*Z* = 4	0.45 × 0.38 × 0.35 mm

### Data collection

**Table:**

Bruker SMART APEX CCD diffractometer	3593 reflections with *I* > 2σ(*I*)
Radiation source: fine-focus sealed tube	*R*_int_ = 0.044
ω scans	θ_max_ = 26.5°, θ_min_ = 2.6°
Absorption correction: multi-scan (SADABS; Bruker, 1998)	*h* = −11→11
*T*_min_ = 0.821, *T*_max_ = 0.928	*k* = −13→13
34596 measured reflections	*l* = −30→30
4807 independent reflections	

### Refinement

**Table:**

Refinement on *F*^2^	Secondary atom site location: difference Fourier map
Least-squares matrix: full	Hydrogen site location: inferred from neighbouring sites
*R*[*F*^2^ > 2σ(*F*^2^)] = 0.047	H atoms treated by a mixture of independent and constrained refinement
*wR*(*F*^2^) = 0.129	*w* = 1/[σ^2^(*F*_o_^2^) + (0.0461*P*)^2^ + 1.5828*P*] where *P* = (*F*_o_^2^ + 2*F*_c_^2^)/3
*S* = 1.03	(Δ/σ)_max_ = 0.001
4807 reflections	Δρ_max_ = 0.26 e Å^−^^3^
301 parameters	Δρ_min_ = −0.29 e Å^−^^3^
0 restraints	Extinction correction: SHELXL2018 (Sheldrick, 2015), Fc^*^=kFc[1+0.001xFc^2^λ^3^/sin(2θ)]^-1/4^
Primary atom site location: structure-invariant direct methods	Extinction coefficient: 0.0135 (11)

### Special details

**Table:**

Geometry. All esds (except the esd in the dihedral angle between two l.s. planes) are estimated using the full covariance matrix. The cell esds are taken into account individually in the estimation of esds in distances, angles and torsion angles; correlations between esds in cell parameters are only used when they are defined by crystal symmetry. An approximate (isotropic) treatment of cell esds is used for estimating esds involving l.s. planes.
Refinement. H atoms were placed at calculated positions in the riding-model approximation, with O—H = 1.00 Å and C—H = 0.93, 0.96 and 0.97 Å for aromatic, methyl and methine H atoms, respectively, and with *U*_iso_(H) = 1.5*U*_eq_(C) for methyl H atoms and 1.2*U*_eq_(C) otherwise.

### Fractional atomic coordinates and isotropic or equivalent isotropic displacement parameters (Å^2^)

**Table:**

	*x*	*y*	*z*	*U*_iso_*/*U*_eq_	
Cl1	1.10374 (7)	0.46682 (6)	1.17331 (2)	0.0588 (2)	
O1	0.67555 (18)	0.23218 (14)	0.99506 (7)	0.0538 (4)	
H1	0.647 (3)	0.2890 (19)	0.9625 (11)	0.081*	
N1	0.65078 (19)	0.43455 (15)	0.93998 (7)	0.0412 (4)	
N2	0.71390 (18)	0.61739 (15)	0.97832 (6)	0.0367 (4)	
C1	0.7255 (2)	0.49090 (18)	0.98494 (8)	0.0374 (4)	
C2	0.6291 (2)	0.64067 (18)	0.92560 (7)	0.0365 (4)	
C3	0.5904 (2)	0.52542 (18)	0.90324 (8)	0.0372 (4)	
C4	0.8032 (2)	0.42008 (18)	1.03298 (8)	0.0369 (4)	
C5	0.7687 (2)	0.29210 (19)	1.03599 (8)	0.0404 (5)	
C6	0.8346 (2)	0.2226 (2)	1.08197 (9)	0.0451 (5)	
H6	0.808858	0.139367	1.084268	0.054*	
C7	0.9371 (2)	0.2747 (2)	1.12404 (8)	0.0453 (5)	
H7	0.980055	0.227497	1.154686	0.054*	
C8	0.9756 (2)	0.3984 (2)	1.12027 (8)	0.0407 (5)	
C9	0.9117 (2)	0.47049 (19)	1.07553 (8)	0.0401 (4)	
H9	0.940636	0.553051	1.073556	0.048*	
C10	0.7779 (2)	0.70811 (17)	1.01924 (7)	0.0343 (4)	
C11	0.7207 (2)	0.7207 (2)	1.06745 (8)	0.0410 (5)	
H11	0.634020	0.678509	1.071702	0.049*	
C12	0.7936 (2)	0.7967 (2)	1.10933 (8)	0.0465 (5)	
H12	0.755755	0.804476	1.142025	0.056*	
C13	0.9217 (2)	0.86161 (19)	1.10368 (8)	0.0431 (5)	
C14	0.9724 (2)	0.8518 (2)	1.05376 (9)	0.0483 (5)	
H14	1.055933	0.897377	1.048605	0.058*	
C15	0.9012 (2)	0.7755 (2)	1.01134 (8)	0.0428 (5)	
H15	0.936377	0.769909	0.978027	0.051*	
C16	1.0063 (3)	0.9367 (2)	1.15138 (10)	0.0616 (7)	
H16A	0.936247	0.989876	1.165958	0.074*	
H16B	1.078251	0.989614	1.137768	0.074*	
C17	1.0880 (4)	0.8561 (3)	1.19769 (13)	0.0940 (11)	
H17A	1.139962	0.907784	1.227008	0.141*	
H17B	1.017021	0.804672	1.211867	0.141*	
H17C	1.159005	0.804410	1.183681	0.141*	
C18	0.6046 (2)	0.76627 (18)	0.90155 (8)	0.0372 (4)	
C19	0.5629 (3)	0.8667 (2)	0.93102 (9)	0.0504 (5)	
H19	0.549954	0.855868	0.967564	0.061*	
C20	0.5404 (3)	0.9826 (2)	0.90656 (11)	0.0641 (7)	
H20	0.513916	1.049426	0.926859	0.077*	
C21	0.5569 (3)	0.9994 (2)	0.85245 (12)	0.0686 (8)	
H21	0.540690	1.077261	0.835939	0.082*	
C22	0.5975 (3)	0.9009 (2)	0.82270 (10)	0.0639 (7)	
H22	0.608119	0.912305	0.785941	0.077*	
C23	0.6226 (2)	0.7851 (2)	0.84696 (9)	0.0475 (5)	
H23	0.651751	0.719374	0.826657	0.057*	
C24	0.4960 (2)	0.48681 (18)	0.85017 (8)	0.0376 (4)	
C25	0.3671 (2)	0.5502 (2)	0.82669 (9)	0.0490 (5)	
H25	0.341256	0.623145	0.843045	0.059*	
C26	0.2763 (3)	0.5057 (3)	0.77900 (10)	0.0602 (6)	
H26	0.190035	0.549194	0.763527	0.072*	
C27	0.3123 (3)	0.3978 (3)	0.75417 (9)	0.0599 (6)	
H27	0.250298	0.368055	0.722302	0.072*	
C28	0.4405 (3)	0.3347 (2)	0.77687 (9)	0.0568 (6)	
H28	0.466097	0.262341	0.760037	0.068*	
C29	0.5316 (3)	0.3779 (2)	0.82458 (8)	0.0471 (5)	
H29	0.617670	0.333929	0.839817	0.057*	

### Atomic displacement parameters (Å^2^)

**Table:**

	*U*^11^	*U*^22^	*U*^33^	*U*^12^	*U*^13^	*U*^23^
Cl1	0.0561 (3)	0.0675 (4)	0.0446 (3)	0.0094 (3)	−0.0121 (2)	−0.0065 (3)
O1	0.0686 (10)	0.0383 (8)	0.0474 (9)	−0.0101 (7)	−0.0079 (7)	0.0039 (7)
N1	0.0487 (10)	0.0358 (9)	0.0348 (8)	−0.0001 (7)	−0.0034 (7)	0.0022 (7)
N2	0.0440 (9)	0.0315 (8)	0.0318 (8)	−0.0011 (7)	−0.0006 (7)	0.0009 (6)
C1	0.0424 (10)	0.0343 (10)	0.0326 (9)	0.0000 (8)	−0.0008 (8)	0.0025 (8)
C2	0.0400 (10)	0.0363 (10)	0.0311 (9)	0.0031 (8)	0.0011 (8)	0.0010 (8)
C3	0.0414 (10)	0.0362 (10)	0.0316 (9)	0.0025 (8)	0.0006 (8)	0.0026 (8)
C4	0.0421 (10)	0.0335 (10)	0.0336 (9)	0.0027 (8)	0.0027 (8)	0.0030 (8)
C5	0.0450 (11)	0.0380 (10)	0.0372 (10)	0.0005 (9)	0.0043 (8)	0.0021 (8)
C6	0.0543 (12)	0.0377 (11)	0.0433 (11)	0.0023 (9)	0.0086 (9)	0.0089 (9)
C7	0.0515 (12)	0.0488 (12)	0.0347 (10)	0.0112 (10)	0.0051 (9)	0.0110 (9)
C8	0.0416 (10)	0.0466 (12)	0.0320 (10)	0.0096 (9)	0.0020 (8)	−0.0016 (9)
C9	0.0441 (11)	0.0364 (10)	0.0370 (10)	0.0052 (8)	−0.0001 (8)	0.0020 (8)
C10	0.0372 (9)	0.0331 (9)	0.0304 (9)	0.0013 (8)	0.0004 (7)	−0.0011 (7)
C11	0.0377 (10)	0.0483 (12)	0.0373 (10)	−0.0013 (9)	0.0076 (8)	0.0021 (9)
C12	0.0518 (12)	0.0558 (13)	0.0321 (10)	0.0068 (10)	0.0083 (9)	−0.0040 (9)
C13	0.0497 (12)	0.0388 (11)	0.0368 (10)	0.0045 (9)	−0.0024 (9)	−0.0023 (9)
C14	0.0479 (12)	0.0486 (12)	0.0478 (12)	−0.0126 (10)	0.0073 (9)	−0.0018 (10)
C15	0.0477 (11)	0.0466 (12)	0.0355 (10)	−0.0050 (9)	0.0109 (9)	−0.0023 (9)
C16	0.0747 (16)	0.0534 (14)	0.0492 (13)	−0.0026 (12)	−0.0083 (12)	−0.0121 (11)
C17	0.113 (3)	0.083 (2)	0.0662 (18)	−0.0077 (19)	−0.0367 (17)	0.0015 (16)
C18	0.0393 (10)	0.0357 (10)	0.0337 (9)	0.0006 (8)	−0.0007 (8)	0.0013 (8)
C19	0.0603 (14)	0.0431 (12)	0.0457 (12)	0.0093 (10)	0.0038 (10)	−0.0019 (10)
C20	0.0767 (17)	0.0389 (12)	0.0701 (17)	0.0139 (12)	−0.0044 (13)	−0.0034 (12)
C21	0.0850 (19)	0.0389 (13)	0.0733 (18)	0.0027 (12)	−0.0085 (14)	0.0181 (12)
C22	0.0799 (17)	0.0586 (15)	0.0495 (14)	−0.0013 (13)	0.0021 (12)	0.0194 (12)
C23	0.0583 (13)	0.0450 (12)	0.0377 (11)	0.0030 (10)	0.0047 (9)	0.0056 (9)
C24	0.0414 (10)	0.0391 (10)	0.0307 (9)	−0.0038 (8)	0.0025 (8)	0.0006 (8)
C25	0.0476 (12)	0.0532 (13)	0.0431 (11)	0.0037 (10)	0.0000 (9)	−0.0028 (10)
C26	0.0478 (13)	0.0774 (18)	0.0484 (13)	0.0038 (12)	−0.0092 (10)	−0.0020 (12)
C27	0.0625 (15)	0.0707 (17)	0.0408 (12)	−0.0156 (13)	−0.0060 (11)	−0.0064 (12)
C28	0.0792 (17)	0.0480 (13)	0.0403 (12)	−0.0074 (12)	0.0030 (11)	−0.0107 (10)
C29	0.0577 (13)	0.0409 (11)	0.0391 (11)	0.0029 (10)	−0.0009 (9)	−0.0013 (9)

### Geometric parameters (Å, º)

**Table:**

Cl1—C8	1.738 (2)	C14—H14	0.9300
O1—C5	1.349 (2)	C15—H15	0.9300
O1—H1	1.00 (3)	C16—C17	1.509 (4)
N1—C1	1.327 (2)	C16—H16A	0.9700
N1—C3	1.372 (2)	C16—H16B	0.9700
N2—C1	1.372 (2)	C17—H17A	0.9600
N2—C2	1.397 (2)	C17—H17B	0.9600
N2—C10	1.441 (2)	C17—H17C	0.9600
C1—C4	1.468 (3)	C18—C23	1.390 (3)
C2—C3	1.375 (3)	C18—C19	1.390 (3)
C2—C18	1.474 (3)	C19—C20	1.382 (3)
C3—C24	1.477 (3)	C19—H19	0.9300
C4—C9	1.403 (3)	C20—C21	1.371 (4)
C4—C5	1.417 (3)	C20—H20	0.9300
C5—C6	1.391 (3)	C21—C22	1.373 (4)
C6—C7	1.373 (3)	C21—H21	0.9300
C6—H6	0.9300	C22—C23	1.381 (3)
C7—C8	1.383 (3)	C22—H22	0.9300
C7—H7	0.9300	C23—H23	0.9300
C8—C9	1.379 (3)	C24—C25	1.383 (3)
C9—H9	0.9300	C24—C29	1.394 (3)
C10—C15	1.376 (3)	C25—C26	1.383 (3)
C10—C11	1.379 (3)	C25—H25	0.9300
C11—C12	1.380 (3)	C26—C27	1.377 (4)
C11—H11	0.9300	C26—H26	0.9300
C12—C13	1.384 (3)	C27—C28	1.372 (4)
C12—H12	0.9300	C27—H27	0.9300
C13—C14	1.386 (3)	C28—C29	1.381 (3)
C13—C16	1.507 (3)	C28—H28	0.9300
C14—C15	1.386 (3)	C29—H29	0.9300
			
C5—O1—H1	109.5	C14—C15—H15	120.5
C1—N1—C3	107.38 (16)	C13—C16—C17	112.5 (2)
C1—N2—C2	107.68 (15)	C13—C16—H16A	109.1
C1—N2—C10	125.28 (15)	C17—C16—H16A	109.1
C2—N2—C10	127.04 (16)	C13—C16—H16B	109.1
N1—C1—N2	109.82 (16)	C17—C16—H16B	109.1
N1—C1—C4	121.54 (17)	H16A—C16—H16B	107.8
N2—C1—C4	128.64 (17)	C16—C17—H17A	109.5
C3—C2—N2	105.22 (16)	C16—C17—H17B	109.5
C3—C2—C18	131.25 (17)	H17A—C17—H17B	109.5
N2—C2—C18	123.34 (17)	C16—C17—H17C	109.5
N1—C3—C2	109.88 (16)	H17A—C17—H17C	109.5
N1—C3—C24	118.18 (17)	H17B—C17—H17C	109.5
C2—C3—C24	131.90 (18)	C23—C18—C19	118.45 (19)
C9—C4—C5	117.94 (17)	C23—C18—C2	118.78 (18)
C9—C4—C1	124.14 (17)	C19—C18—C2	122.77 (18)
C5—C4—C1	117.91 (17)	C20—C19—C18	120.6 (2)
O1—C5—C6	117.42 (18)	C20—C19—H19	119.7
O1—C5—C4	122.68 (17)	C18—C19—H19	119.7
C6—C5—C4	119.88 (19)	C21—C20—C19	120.2 (2)
C7—C6—C5	121.2 (2)	C21—C20—H20	119.9
C7—C6—H6	119.4	C19—C20—H20	119.9
C5—C6—H6	119.4	C20—C21—C22	119.8 (2)
C6—C7—C8	119.18 (18)	C20—C21—H21	120.1
C6—C7—H7	120.4	C22—C21—H21	120.1
C8—C7—H7	120.4	C21—C22—C23	120.5 (2)
C9—C8—C7	121.29 (19)	C21—C22—H22	119.8
C9—C8—Cl1	118.69 (17)	C23—C22—H22	119.8
C7—C8—Cl1	120.00 (15)	C22—C23—C18	120.4 (2)
C8—C9—C4	120.42 (19)	C22—C23—H23	119.8
C8—C9—H9	119.8	C18—C23—H23	119.8
C4—C9—H9	119.8	C25—C24—C29	118.41 (19)
C15—C10—C11	120.72 (18)	C25—C24—C3	122.32 (19)
C15—C10—N2	119.52 (17)	C29—C24—C3	119.12 (18)
C11—C10—N2	119.63 (17)	C26—C25—C24	120.3 (2)
C10—C11—C12	119.32 (19)	C26—C25—H25	119.8
C10—C11—H11	120.3	C24—C25—H25	119.8
C12—C11—H11	120.3	C27—C26—C25	120.8 (2)
C11—C12—C13	121.36 (19)	C27—C26—H26	119.6
C11—C12—H12	119.3	C25—C26—H26	119.6
C13—C12—H12	119.3	C28—C27—C26	119.4 (2)
C12—C13—C14	118.03 (19)	C28—C27—H27	120.3
C12—C13—C16	120.6 (2)	C26—C27—H27	120.3
C14—C13—C16	121.3 (2)	C27—C28—C29	120.4 (2)
C13—C14—C15	121.4 (2)	C27—C28—H28	119.8
C13—C14—H14	119.3	C29—C28—H28	119.8
C15—C14—H14	119.3	C28—C29—C24	120.7 (2)
C10—C15—C14	119.03 (19)	C28—C29—H29	119.6
C10—C15—H15	120.5	C24—C29—H29	119.6
			
C3—N1—C1—N2	0.4 (2)	C15—C10—C11—C12	−3.6 (3)
C3—N1—C1—C4	179.45 (17)	N2—C10—C11—C12	172.22 (18)
C2—N2—C1—N1	−1.1 (2)	C10—C11—C12—C13	0.8 (3)
C10—N2—C1—N1	178.44 (17)	C11—C12—C13—C14	2.3 (3)
C2—N2—C1—C4	179.94 (19)	C11—C12—C13—C16	−175.5 (2)
C10—N2—C1—C4	−0.5 (3)	C12—C13—C14—C15	−2.6 (3)
C1—N2—C2—C3	1.3 (2)	C16—C13—C14—C15	175.1 (2)
C10—N2—C2—C3	−178.22 (17)	C11—C10—C15—C14	3.3 (3)
C1—N2—C2—C18	−174.15 (18)	N2—C10—C15—C14	−172.56 (18)
C10—N2—C2—C18	6.3 (3)	C13—C14—C15—C10	−0.1 (3)
C1—N1—C3—C2	0.4 (2)	C12—C13—C16—C17	71.3 (3)
C1—N1—C3—C24	−177.67 (17)	C14—C13—C16—C17	−106.4 (3)
N2—C2—C3—N1	−1.1 (2)	C3—C2—C18—C23	−40.4 (3)
C18—C2—C3—N1	173.9 (2)	N2—C2—C18—C23	133.8 (2)
N2—C2—C3—C24	176.7 (2)	C3—C2—C18—C19	139.0 (2)
C18—C2—C3—C24	−8.4 (4)	N2—C2—C18—C19	−46.8 (3)
N1—C1—C4—C9	164.28 (19)	C23—C18—C19—C20	−0.2 (3)
N2—C1—C4—C9	−16.9 (3)	C2—C18—C19—C20	−179.6 (2)
N1—C1—C4—C5	−14.4 (3)	C18—C19—C20—C21	1.0 (4)
N2—C1—C4—C5	164.4 (2)	C19—C20—C21—C22	−0.7 (4)
C9—C4—C5—O1	−174.41 (19)	C20—C21—C22—C23	−0.4 (4)
C1—C4—C5—O1	4.4 (3)	C21—C22—C23—C18	1.1 (4)
C9—C4—C5—C6	4.1 (3)	C19—C18—C23—C22	−0.8 (3)
C1—C4—C5—C6	−177.12 (19)	C2—C18—C23—C22	178.6 (2)
O1—C5—C6—C7	176.40 (19)	N1—C3—C24—C25	140.6 (2)
C4—C5—C6—C7	−2.2 (3)	C2—C3—C24—C25	−37.0 (3)
C5—C6—C7—C8	−0.3 (3)	N1—C3—C24—C29	−34.8 (3)
C6—C7—C8—C9	0.9 (3)	C2—C3—C24—C29	147.5 (2)
C6—C7—C8—Cl1	179.40 (16)	C29—C24—C25—C26	0.1 (3)
C7—C8—C9—C4	1.1 (3)	C3—C24—C25—C26	−175.4 (2)
Cl1—C8—C9—C4	−177.38 (15)	C24—C25—C26—C27	0.1 (4)
C5—C4—C9—C8	−3.6 (3)	C25—C26—C27—C28	−0.6 (4)
C1—C4—C9—C8	177.71 (19)	C26—C27—C28—C29	0.8 (4)
C1—N2—C10—C15	107.0 (2)	C27—C28—C29—C24	−0.6 (4)
C2—N2—C10—C15	−73.5 (3)	C25—C24—C29—C28	0.1 (3)
C1—N2—C10—C11	−68.8 (3)	C3—C24—C29—C28	175.8 (2)
C2—N2—C10—C11	110.7 (2)		

### Hydrogen-bond geometry (Å, º)

*Cg* is the centroid of the C4–C9 aryl ring.

**Table:**

*D*—H···*A*	*D*—H	H···*A*	*D*···*A*	*D*—H···*A*
O1—H1···N1	1.00	1.66	2.549 (1)	145
C19—H19···O1^i^	0.93	2.57	3.242 (3)	129
C15—H15···*Cg*^ii^	0.93	2.72	3.527 (2)	146

Symmetry codes: (i) −*x*+1, −*y*+1, −*z*+2; (ii) −*x*+2, −*y*+1, −*z*+2.
