# Supplementary figures and images for: 4-Chloro-2-[1-(4-ethyl­phen­yl)-4,5-diphenyl-1H-imidazol-2-yl]phenol
Source: IUCrdata. 2020 Jan 3;5(Pt 1):x191690. doi: 10.1107/S2414314619016900 (PMC9462148; doi:10.1107/S2414314619016900)

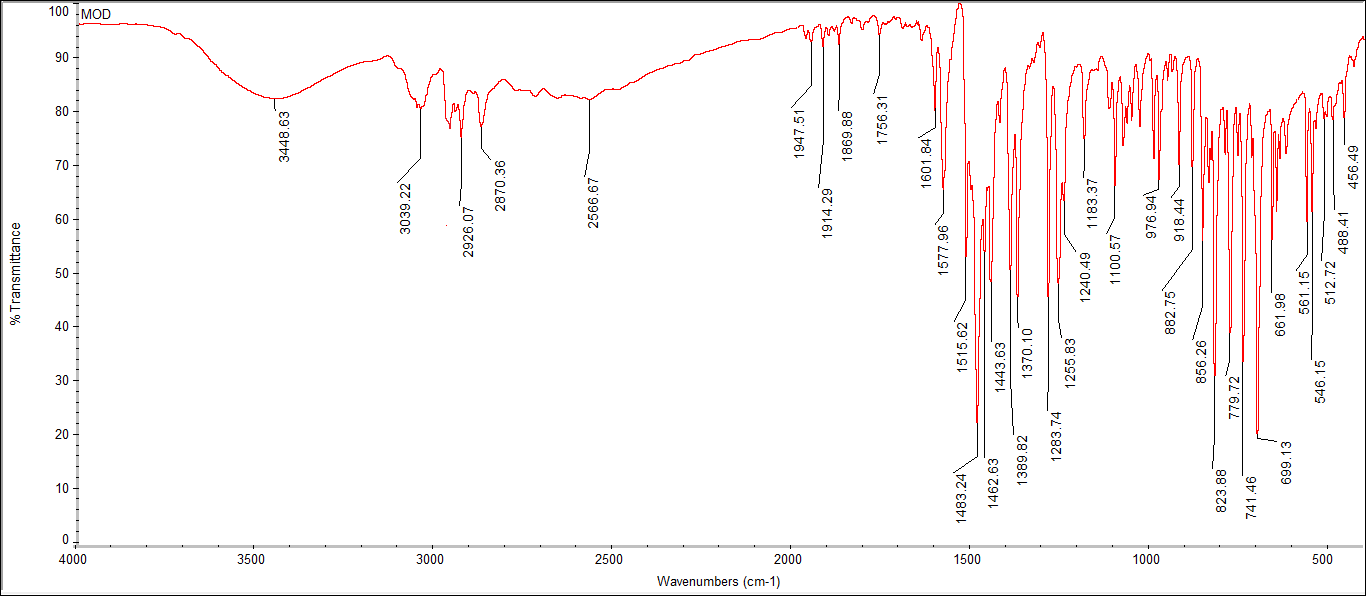

Supplement: Supplementary file 3 [file x-05-x191690-sup3.tif]

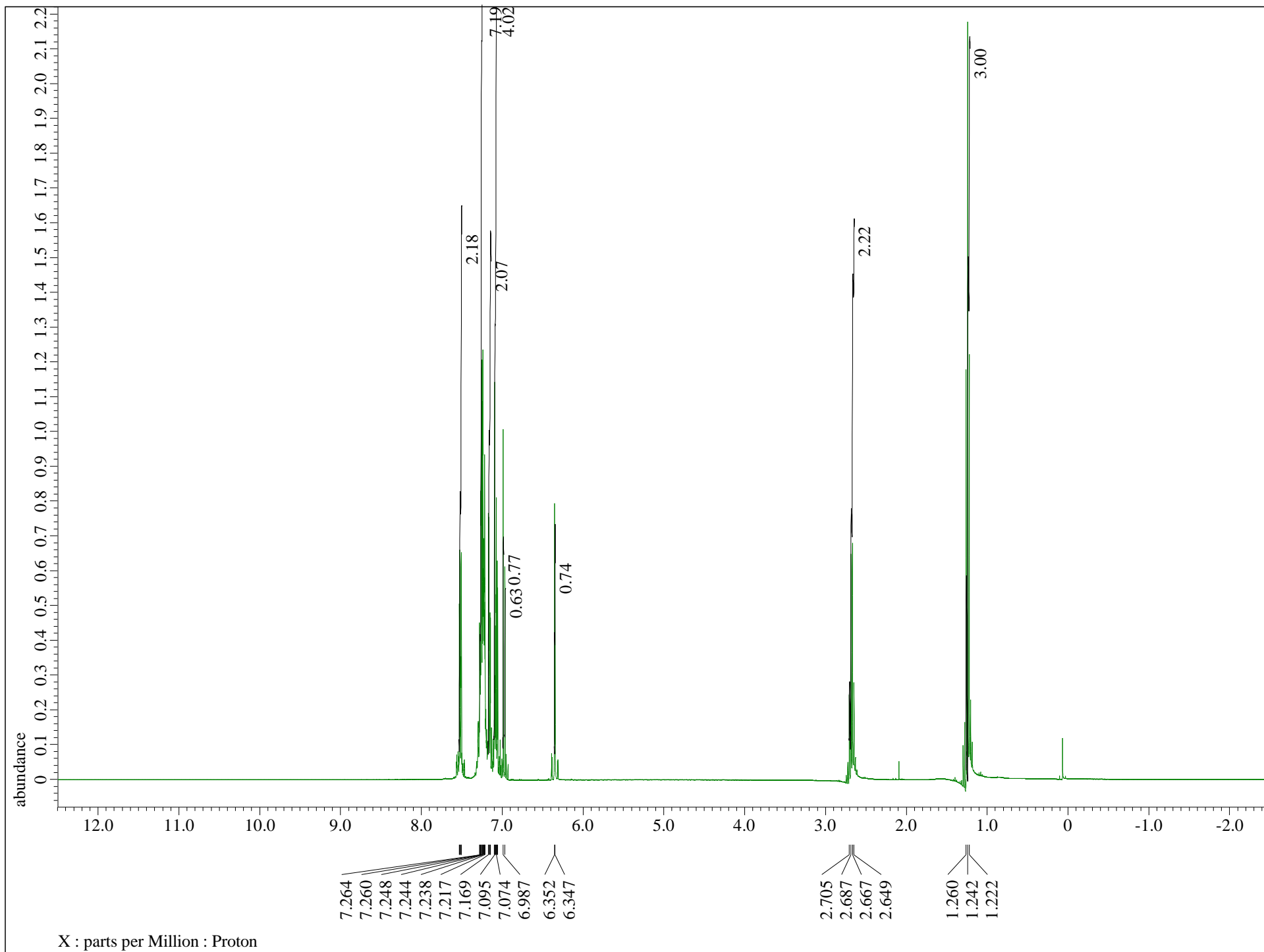

Supplement: Supplementary file 5 [file x-05-x191690-sup5.pdf]
