# Supplementary material for: 4-Chloro-2-[1-(4-ethyl­phen­yl)-4,5-diphenyl-1H-imidazol-2-yl]phenol
Source: IUCrdata. 2020 Jan 3;5(Pt 1):x191690. doi: 10.1107/S2414314619016900 (PMC9462148; doi:10.1107/S2414314619016900)

KARNATAK UNIVERSITY
UNIVERSITY SCIENCE INSTRUMENTS CENTRE
DHARWAD

Sample Informatior

Analyzed by	: Admin
Analyzed	: 11/23/2017 4:41:19 PM
Sample Type	: Unknown
Level #	: 1
Sample Name	: MoD
Sample ID	:
IS Amount	: [1]=1

Spectrum

Line#:1 R.Time:3.8(Scan#:459)
MassPeaks:396
RawMode:Single 3.8(459) BasePeak:450(1910473)
BG Mode:None Group 1 - Event 1

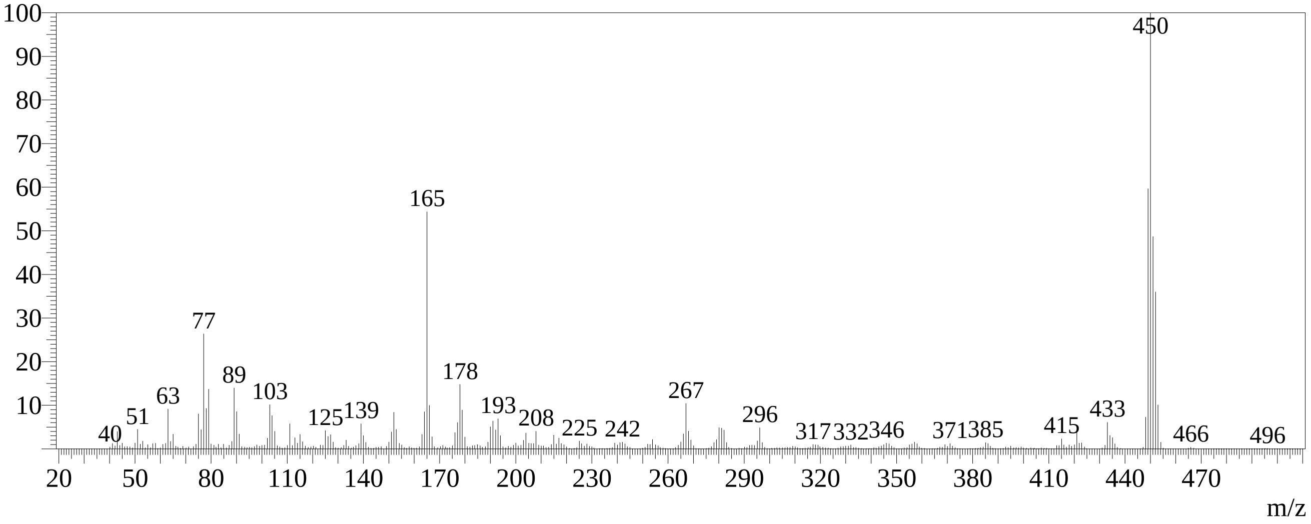

Supplement: Supplementary file 4 [file x-05-x191690-sup4.pdf]
